# Comparative biochemical kinase activity analysis identifies rivoceranib as a highly selective VEGFR2 inhibitor

**DOI:** 10.1007/s00280-023-04534-7

**Published:** 2023-05-06

**Authors:** Seong Jang, Bill Strickland, Lynda Finis, Jeffrey J. Kooijman, Janneke J. T. M. Melis, Guido J. R. Zaman, Jan Van Tornout

**Affiliations:** 1 Elevar Therapeutics, Fort Lee, NJ USA; 2 Oncolines B.V., Oss, The Netherlands

**Keywords:** VEGFR2, Angiogenesis, Oral kinase inhibitor, Rivoceranib, Comparative kinase activity

## Abstract

Vascular endothelial growth factor receptor 2 (VEGFR2), a key regulator of tumor angiogenesis, is highly expressed across numerous tumor types and has been an attractive target for anti-cancer therapy. However, clinical application of available VEGFR2 inhibitors has been challenged by limited efficacy and a wide range of side effects, potentially due to inadequate selectivity for VEGFR2. Thus, development of potent VEGFR2 inhibitors with improved selectivity is needed. Rivoceranib is an orally administered tyrosine kinase inhibitor that potently and selectively targets VEGFR2. A comparative understanding of the potency and selectivity of rivoceranib and approved inhibitors of VEGFR2 is valuable to inform rationale for therapy selection in the clinic. Here, we performed biochemical analyses of the kinase activity of VEGFR2 and of a panel of 270 kinases to compare rivoceranib to 10 FDA-approved kinase inhibitors (“reference inhibitors”) with known activity against VEGFR2. Rivoceranib demonstrated potency within the range of the reference inhibitors, with a VEGFR2 kinase inhibition IC_50_ value of 16 nM. However, analysis of residual kinase activity of the panel of 270 kinases showed that rivoceranib displayed greater selectivity for VEGFR2 compared with the reference inhibitors. Differences in selectivity among compounds within the observed range of potency of VEGFR2 kinase inhibition are clinically relevant, as toxicities associated with available VEGFR2 inhibitors are thought to be partly due to their effects against kinases other than VEGFR2. Together, this comparative biochemical analysis highlights the potential for rivoceranib to address clinical limitations associated with off-target effects of currently available VEGFR2 inhibitors.

**Supplementary Information:**

The online version contains supplementary material available at 10.1007/s00280-023-04534-7.

## Introduction

Angiogenesis, the formation of new blood vessels from existing vasculature, is a well-known hallmark of cancer and is considered essential for tumor progression and metastasis [1, 2]. By enabling a supply of oxygen, nutrients, and growth factors to the tumor microenvironment as well as an avenue for tumor dissemination to distant sites, angiogenesis contributes to tumor growth, metastasis, and drug resistance across many tumor types [3]. Angiogenesis is driven by the binding of vascular endothelial growth factor (VEGF), secreted by tumor cells and surrounding stroma, to VEGF receptor 2 (VEGFR2; also known as KDR). VEGFR2 is the main signaling receptor for VEGF and is expressed on endothelial cells [4]. Elevated VEGF mRNA expression has been detected in a variety of tumors, and this increased expression can serve as an important prognostic indicator in patients with cancer [5, 6]. VEGF-mediated activation of VEGFR2 leads to the proliferation and survival of endothelial cells, resulting in the formation of new blood vessels. VEGFR2 is thus a key receptor regulating angiogenic function, and agents targeting VEGFR2 are actively pursued as anti-cancer therapies.

Both antibodies and small molecules with activity against VEGFR2 have been approved for the treatment of a variety of tumor types. Bevacizumab, a recombinant humanized monoclonal antibody targeting VEGF, was the first approved therapy targeting the VEGF–VEGFR2 axis for the treatment of cancer and has demonstrated beneficial clinical effects in a variety of tumor types [2, 7, 8]. However, anti-VEGF antibodies have been associated with high immunogenicity and low stability. The clinical application of these antibodies is further limited by the effects associated with the inhibition of physiological angiogenesis [2]. Inhibition of tumor angiogenesis via targeting VEGFRs has achieved greater success than via targeting VEGF. Ramucirumab, a VEGFR2-targeted monoclonal antibody, and several VEGFR small molecule inhibitors have been approved for clinical use [2, 9]. However, ramucirumab has been associated with limited efficacy, potentially severe side effects, and high cost [10, 11]. Similarly, currently available small molecules have been associated with acquired drug resistance, limited efficacy, compound toxicity, and a wide range of side effects [12]. Toxicities of VEGFR inhibitors observed in the clinic include hypertension, proteinuria, hypothyroidism, leukoencephalopathy syndrome, and arterial thrombosis, among others [2]. The toxic effects of available VEGFR inhibitors may be due to the high structural similarity of VEGFR protein family members and other tyrosine kinases (including PDGFRs, CSF1R, FLT-3, and c-Kit, among others) and the lack of selectivity of available small molecule inhibitors for binding to VEGFR2 [12]. Therefore, further investigation of more selective VEGFR2 inhibitors is needed.

Rivoceranib, a highly potent inhibitor of VEGFR2, was the first small molecule tyrosine kinase inhibitor to be approved in gastric cancer under the name apatinib in China in December 2014, and has demonstrated promising anti-tumor activity in a variety of tumor types, including hepatocellular carcinoma, breast cancer, and non-small cell lung cancer [13–16]. Through binding to the intracellular ATP-binding domain of VEGFR2, apatinib has been shown to block VEGF-mediated downstream signaling, endothelial cell proliferation, and tumor angiogenesis [17]. In addition, apatinib has demonstrated the capacity to induce apoptosis and autophagy as well as reverse multidrug resistance [18, 19]. Rivoceranib is currently being developed in the US as monotherapy and in combination with chemotherapy or immunotherapy in clinical trials across multiple tumor types, including gastric cancer, hepatocellular carcinoma, adenoid cystic carcinoma, and colorectal cancer. Rivoceranib has been well tolerated in clinical trials worldwide with manageable toxicities, and the majority of adverse events were mild to moderate in severity [20].

Given the clinical challenges thought to be associated with the variable selectivity of current small molecule inhibitors of VEGFR2, understanding and comparison of the potency and selectivity of these agents are important to inform therapy selection in the clinic. Here, we performed head-to-head biochemical analyses of rivoceranib and 10 additional FDA-approved TKIs with known activity against VEGFR2 (Table 1) to compare their activity and selectivity against VEGFR2 and a panel of 270 kinases (representing approximately half of the total number of known kinases) to serve as a potential basis for clinical decision-making.

**Table 1:** Small molecule kinase inhibitors with activity against VEGFR2 included in the current study

Inhibitor	Initial FDA approval date*	Current indication*	Primary targets
Axitinib	2012	Advanced renal cell carcinoma	VEGFR1-3
Cabozantinib	2016 2019 2021	Advanced renal cell carcinoma Hepatocellular carcinoma Differentiated thyroid cancer	MET, RET, AXL, VEGFR2, FLT3, c-KIT
Lenvatinib	2015 2016 2018 2021	Differentiated thyroid cancer Advanced renal cell carcinoma Hepatocellular carcinoma Advanced endometrial carcinoma (in combination with pembrolizumab)	VEGFR1-3, PDGFRα, FGFR, KIT, RET
Nintedanib	2014 2019	Idiopathic pulmonary fibrosis Interstitial lung disease	VEGFR1-3, PDGFRα, FGFR1-3
Pazopanib	2009 2012	Advanced renal cell carcinomar Advanced soft tissue sarcoma	VEGFR1-3, PDGFRα, FGFR1-3, KIT
Regorafenib	2012 2013 2017	Advanced colorectal cancer Advanced gastrointestinal stromal tumors Hepatocellular carcinoma	VEGFR1-3, TIE2, KIT, RET, RAF1, BRAF, PDGFR, FGFR
Sorafenib	2005 2007 2013	Advanced renal cell carcinoma Hepatocellular carcinoma Metastatic differentiated thyroid cancer	RAF-1, VEGR1-3, PDGFR-β, FLT-3, c-KIT
Sunitinib	2006 2011	Gastrointestinal stromal tumors, advanced renal cell carcinoma Progressive neuroendocrine cancerous tumors located in the pancreas	VEGFR1-3, PDGFR, c-KIT, FLT-3, CSF1R, RET
Tivozanib	2021	Relapsed or refractory advanced renal cell carcinoma	VEGFR1-3, PDGFRα/β, c-KIT
Vandetanib	2011	Advanced medullary thyroid cancer	VEGFR-2, EGFR, RET
Rivoceranib	Investigational in U.S.; Approved as Apatinib in China (2014)	In China: Late-stage gastric carcinoma	VEGFR-2, c-KIT, c-SRC

^*^FDA approval history and indication information obtained from www.drugs.com/history. Accessed September 30, 2022

## Methods

### VEGFR2 inhibitors

A panel of 10 FDA-approved kinase inhibitors with known activity against VEGFR2 (“reference inhibitors”) plus the investigational VEGFR2 inhibitor rivoceranib was evaluated in this study (Table 1). All VEGFR2 reference inhibitors were acquired from commercial vendors and stored as powder at 4 °C. Rivoceranib was obtained from Elevar Therapeutics. Prior to use in experiments, compounds were dissolved in 100% dimethyl sulfoxide (DMSO), then further diluted with assay buffer to prepare the final test compound solution.

### Binding assay

The affinity of rivoceranib for VEGFR2 was determined by surface plasmon resonance (SPR) using biotinylated VEGFR2 and Biacore T200 [21]. Biotinylated recombinant cytoplasmic domain of VEGFR2, encompassing amino acid residue 790 to 1356 (C-terminus), was purchased from Carna Biosciences, Inc. (Kobe, Japan) (Cat. No. 08–491-20N). Biotinylated VEGFR2 cytoplasmic domain was diluted to 40 µg/mL in pre-cooled Biacore buffer (50 mM Tris pH 7.5, 0.05% Tween 20, 150 mM NaCl and 5 mM MgCl_2_) and immobilized with the biotin tag on a streptavidin-coated chip (Cat. No. BR100531; Cytiva). First, the streptavidin on the sensor chip was washed three times with a washing solution (1 M NaCl, 50 mM NaOH) for a period of 60 s with a flow rate of 10 µL/min. Then, biotinylated protein was captured on the chip with a targeted immobilization level of 4000 RU. Remaining streptavidin was blocked with 10 µg/mL biocytin injections to circumvent a specific binding of compound to the streptavidin-bound surface. Immobilization was performed at 4 °C. Subsequent binding assays were conducted at 22 °C. Rivoceranib was dissolved to a 10 mM solution in 100% DMSO and diluted in Biacore buffer without DMSO to obtain a solution with 1% DMSO. Further dilutions were made in Biacore buffer containing 1% DMSO, which was also used as running buffer during the experiments. The kinetic constants of rivoceranib were determined with single-cycle kinetics with five consecutive injections with an increasing compound concentration gradient of 10–31.6–100–316–1000 nM. Experiments were performed with a flow rate of 30 µl/min, an association time of 100 s per concentration and dissociation period of 1200 s. A blank run was performed before the compound range was injected. This blank run is an injection range with five times a buffer injection with the same flow rate, contact time, and dissociation time. The sensorgrams were analyzed with SPR Evaluation Software using the method of double referencing. In the first step, the reference channel was subtracted from the channel containing immobilized protein, and then the reference curve obtained with buffer injections was subtracted. The resulting curve was fitted with a 1:1 binding model. The kinetic constants (*k*_a_, *k*_d_) were geometrically averaged from two experimental replicates each with two technical replicates. The equilibrium dissociation constant, that is, the binding affinity (*K*_D_), was calculated from the ratio of the association and dissociation rate constant (*K*_D_ = *k*_d_/*k*_a_).

### Enzyme activity assays

Kinase assays were performed to evaluate inhibitor activity on VEGFR2 and other kinases using either mobility shift assays (MSA) or immobilized metal ion affinity particle (IMAP) assays at Carna Biosciences, Inc (Kobe, Japan). The panel of kinases assayed comprises all wild-type kinases available for profiling at Carna Biosciences, Inc. For VEGFR2, the same cytoplasmic domain was used as in the SPR binding assay. For dose–response and determination of the half-maximum inhibitory concentration (IC_50_), rivoceranib was tested in duplicate 10-point dilution series (Supplemental Table 1). For determination of the percent inhibition of 270 wild-type kinases, rivoceranib was tested at two fixed concentrations (160 nM and 1600 nM). For the ten reference inhibitors, the VEGFR2 IC_50_ values and percent inhibition at 1000 nM were previously determined in identical assays [22, 23].

Off-chip MSA was performed by preparing a 4 × substrate/ATP/metal solution using kit buffer (20 mM HEPES, 0.01% Triton X-100, 5 mM DTT, pH 7.5), and a 2 × kinase solution was prepared in assay buffer (20 mM HEPES, 0.01% TritonX-100, 1 mM DTT, pH 7.5). The VEGFR2 substrate was used at a concentration of 1000 nM and ATP at a concentration of 75 µmol/L, which corresponds to the K_m,ATP_ of the enzyme. A 4 × compound solution and the 4 × substrate/ATP/metal solution, and 2 × kinase solution were mixed and incubated in a 384-well microplate at room temperature for 1 or 5 h, dependent on the kinase. Termination Buffer (QuickScout Screening Assist MSA; Carna Biosciences) was added to the well, and the reaction mixture was applied to the LabChip™ system (Perkin Elmer) to separate and quantitate the product and substrate peptide peaks. The kinase reaction was measured using the product ratio calculated from the peak heights of the product (P) and substrate (S) peptides: [P/(P + S)].

For the IMAP assays, a 4 × substrate/ATP/metal solution and 2 × kinase solution were prepared using assay buffer (20 mM HEPES, 0.01% Tween 20, 2 mM DTT, pH 7.4). ATP concentrations in the solution were within twofold of the affinity for ATP (K_M,ATP_) for each individual kinase. Then, the 4 × compound solution, 4 × substrate/ATP/metal solution, and 2 × kinase solution were mixed and incubated in a single well of a polystyrene 384-well black microplate. After incubating for 1 h at room temperature, IMAP-binding reagent (IMAP Screening Express kit; Molecular Devices) was added to the well and incubated for 30 min. The kinase reaction in each well was measured via fluorescence polarization at excitation of 485 nm and emission of 530 nm.

The value obtained from the reaction control (complete reaction mixture) was set as 0% inhibition, and the background value (mixture lacking enzyme) was set as 100% inhibition to calculate the percent inhibition for each test solution. IC_50_ values were calculated by fitting a 4-parameter logistic curve to the percentage inhibition values at the tested concentrations. For the determination of the percent inhibition at fixed inhibitor concentration, percentage inhibition values < 0 were capped at 0, while values > 100 were capped at 100. The capped percentage inhibition values were converted to residual kinase activity values (100 % inhibition), and residual kinase activity values were visualized via radar chart.

### Kinome tree biochemical selectivity

Using the data obtained from the kinase activity assays described above, the percentage inhibition values for each inhibitor were mapped to the kinome tree. Protein names as reported by Carna could be mapped to HGNC names for 266 of the 270 kinases on which all inhibitors were profiled. A list of the kinases assayed is provided in the Supplemental Methods. Different protein isoforms or complexes of the same kinase were profiled; only one was mapped to the HGNC name used in the kinome tree. Proteins not mapped to HGNC name included LYNb, AurA/TPX2, CDK2/CycE1, and PKCβ2. For each inhibitor and kinase pair, the percentage inhibition value was grouped into one of the following four categories: > 95%; > 90% & ≤ 95%; > 50% & ≤ 90%; and ≤ 50%. CORAL was used to generate the kinome trees [24].

## Results

### Rivoceranib binds to VEGFR2 with low nanomolar affinity

**Fig. 1:**
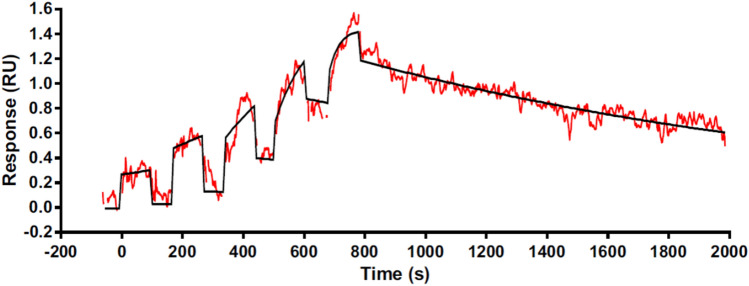
Representative sensorgram of a single-cycle kinetics experiment with rivoceranib and the cytoplasmic domain of VEGFR2. The red line corresponds to the actual response and the black line to the response after fitting with a 1:1 model using Biacore T200 software. Based on four replicates, a *K*_D_ of 2.08 nM was determined (Supplemental Table 2)

The binding affinity (*K*_D_) of rivoceranib to VEGFR2 was determined using SPR, a label-free and probe-free method for the determination of kinetics of biomolecular interactions. The advantage of SPR over kinase activity assays is that the kinetics of binding is observed in real time and no confounding competitive molecules, such as ATP or substrates, are present in the reaction. For SPR, the target protein is immobilized on a chip, and a solution of the kinase inhibitor is injected and flown over the surface. Binding of a compound changes the refractive index and is measured as a positive signal expressed in resonance units (Fig. 1). Dissociation of the compound results in a decrease of resonance units. The resulting sensorgram shows the rate of formation of a target–compound complex and the rate of its subsequent dissociation.

A recombinant, insect cell expressed protein corresponding to the cytoplasmic domain of VEGFR2 and encompassing the tyrosine kinase domain of the receptor was used to study the binding kinetics of rivoceranib. The kinase was immobilized on a streptavidin-coated chip using a biotin tag. Figure 1 shows a representative sensorgram of rivoceranib binding. The *K*_D_, calculated from the association rate (*k*_a_) and dissociation rate constant (*k*_d_), was 2 nM (Supplemental Table 2).

### VEGFR2 kinase inhibition is similar between rivoceranib and reference inhibitors

**Fig. 2:**
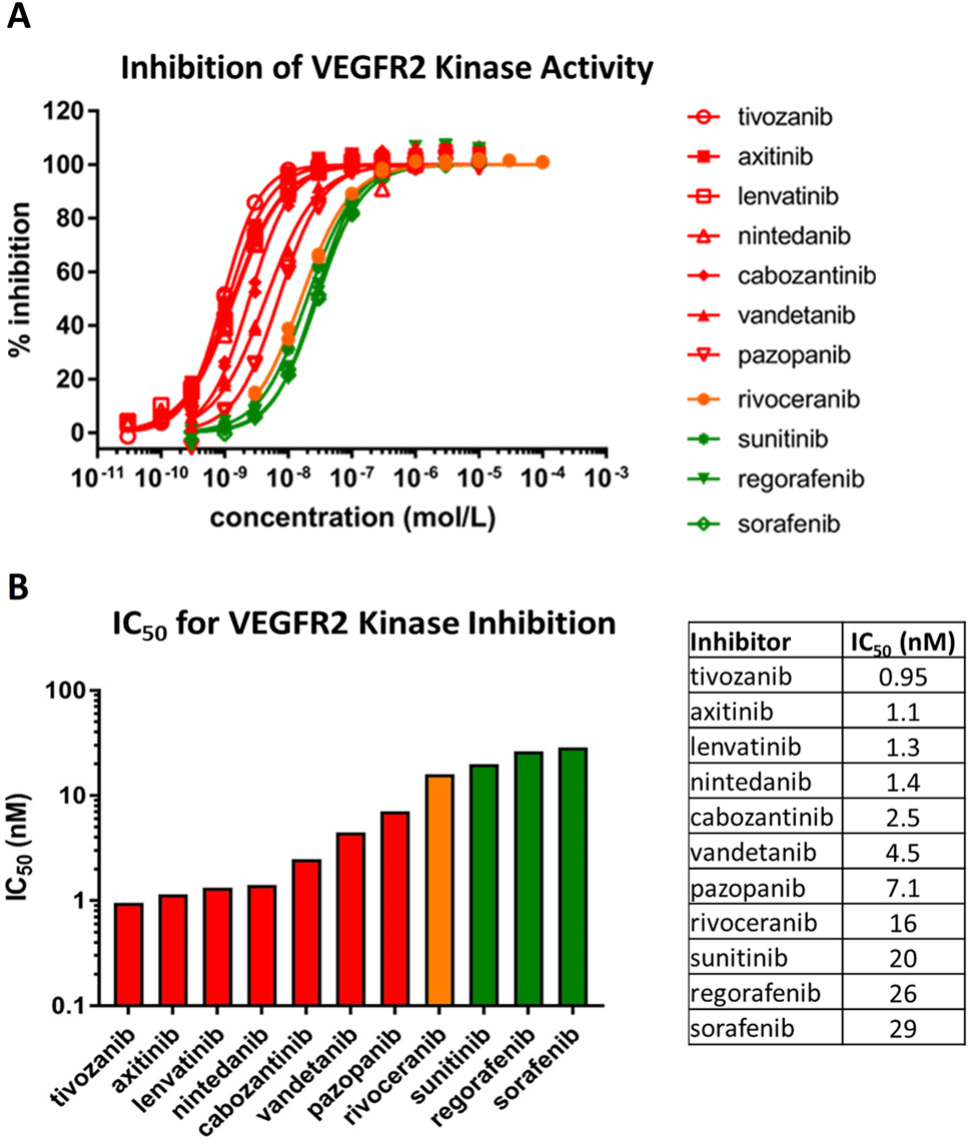
Potency of rivoceranib-mediated VEGFR2 kinase inhibition is within the range of reference inhibitors. Off-shift mobility shift assays (MSA) were performed to measure VEGFR2 kinase activity in the presence of increasing concentrations of rivoceranib and 10 FDA-approved inhibitors. **A** Dose-response curves for extent of inhibition of VEGFR2 kinase activity mediated by each inhibitor. Rivoceranib displayed in orange: inhibitors with greater potency displayed in red, and those with less potency displayed in green. **B** IC_50_ values obtained from dose-response curves of VEGFR2 kinase inhibition mediated by each inhibitor. Inhibitors were ordered from most potent (left) to least potent (right)

The same cytoplasmic domain fragment of VEGFR2 was used to determine the inhibitory potency (IC_50_) of rivoceranib in an enzyme activity assay using a MSA. In MSA, the phosphorylation of a peptide substrate is quantitatively measured through the effect of phosphorylation on electrophoretic mobility of the peptide. The assay was performed at an ATP concentration of 75 µM, which corresponds to the *K*_m,ATP_ of the recombinant enzyme (74 µM). The activity of rivoceranib in the kinase assay was compared to that of 10 FDA-approved TKIs (“reference inhibitors”) with known activity against VEGFR2, which were measured in the same MSA. As shown in Fig. 2A, while some variation in potency of VEGFR2 kinase activity inhibition was observed between compounds tested, rivoceranib activity against VEGFR2 was within the range of the inhibition mediated by the reference inhibitors. IC_50_ values calculated based on the concentration–percent inhibition curves ranged from 0.95 nM to 29 nM for the reference inhibitors, and the IC_50_ value for rivoceranib was 16 nM (Fig. 2B). Four inhibitors (tivozanib, axitinib, lenvatinib, and nintedanib) demonstrated approximately tenfold lower IC_50_ values than rivoceranib, three inhibitors (cabozantinib, vandetanib, and pazopanib) demonstrated approximately twofold to sixfold lower IC_50_ values, and three inhibitors (sunitinib, regorafenib, and sorafenib) demonstrated higher IC_50_ values. Tivozanib was the most potent VEGFR2 inhibitor, while sorafenib was the least potent. Together, these data demonstrate that rivoceranib mediates VEGFR2 kinase inhibition within the range of that seen with the reference inhibitors.

### Rivoceranib retains greater overall activity of non-targeted kinases compared with reference inhibitors

**Fig. 3:**
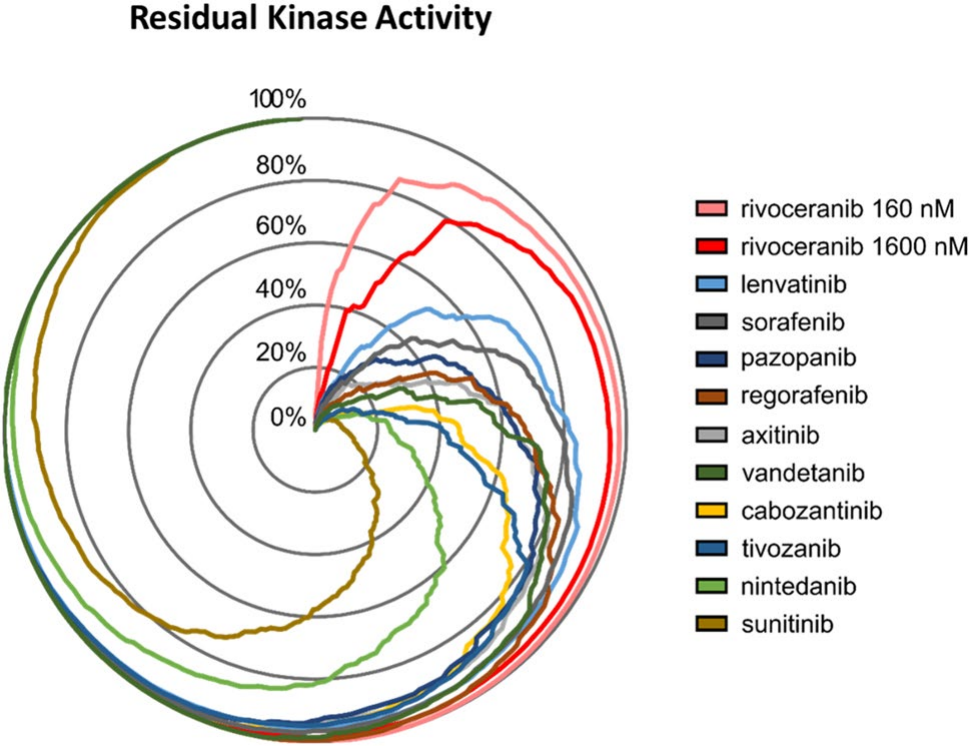
Rivoceranib maintains greater overall residual activity of 270 kinases compared with reference inhibitors. The activity of 270 kinases was measured in the presence of a fixed concentration of each inhibitor (160 nM and 1600 nM rivoceranib, and 1000 nM for all reference inhibitors), and the percent inhibition values were calculated for each kinase + inhibitor pair. Residual kinase activity values were calculated as 100 % inhibition and visualized via radar chart

To compare the selectivity of rivoceranib and the 10 reference inhibitors, we performed either IMAP assays or off-chip MSA to assess the residual kinase activity of a panel of 270 kinases in the presence of 160 nM or 1600 nM rivoceranib or 1000 nM of reference inhibitor. The 270 kinases represent a broad representation of the more than 500 kinases encoded by the human genome. The rivoceranib concentrations tested represent values that are tenfold and 100-fold higher than the IC_50_ of rivoceranib in the VEGFR2 kinase activity assay. As shown in Fig. 3, radar chart visualization of these results demonstrates substantial differences in the overall residual activity across the panel of kinases between inhibitors. Among all of the inhibitors profiled, rivoceranib demonstrated the greatest residual kinase activity (at both 160 nM and 1600 nM) across the panel of kinases. Sunitinib demonstrated the least residual kinase activity. When many kinases retain high residual kinase activity in the presence of an inhibitor, the inhibitor is considered more selective, suggesting that rivoceranib has greater selectivity compared with the reference inhibitors.

### Rivoceranib is the most selective inhibitor of VEGFR2 kinase activity among the tested inhibitors

**Fig. 4:**
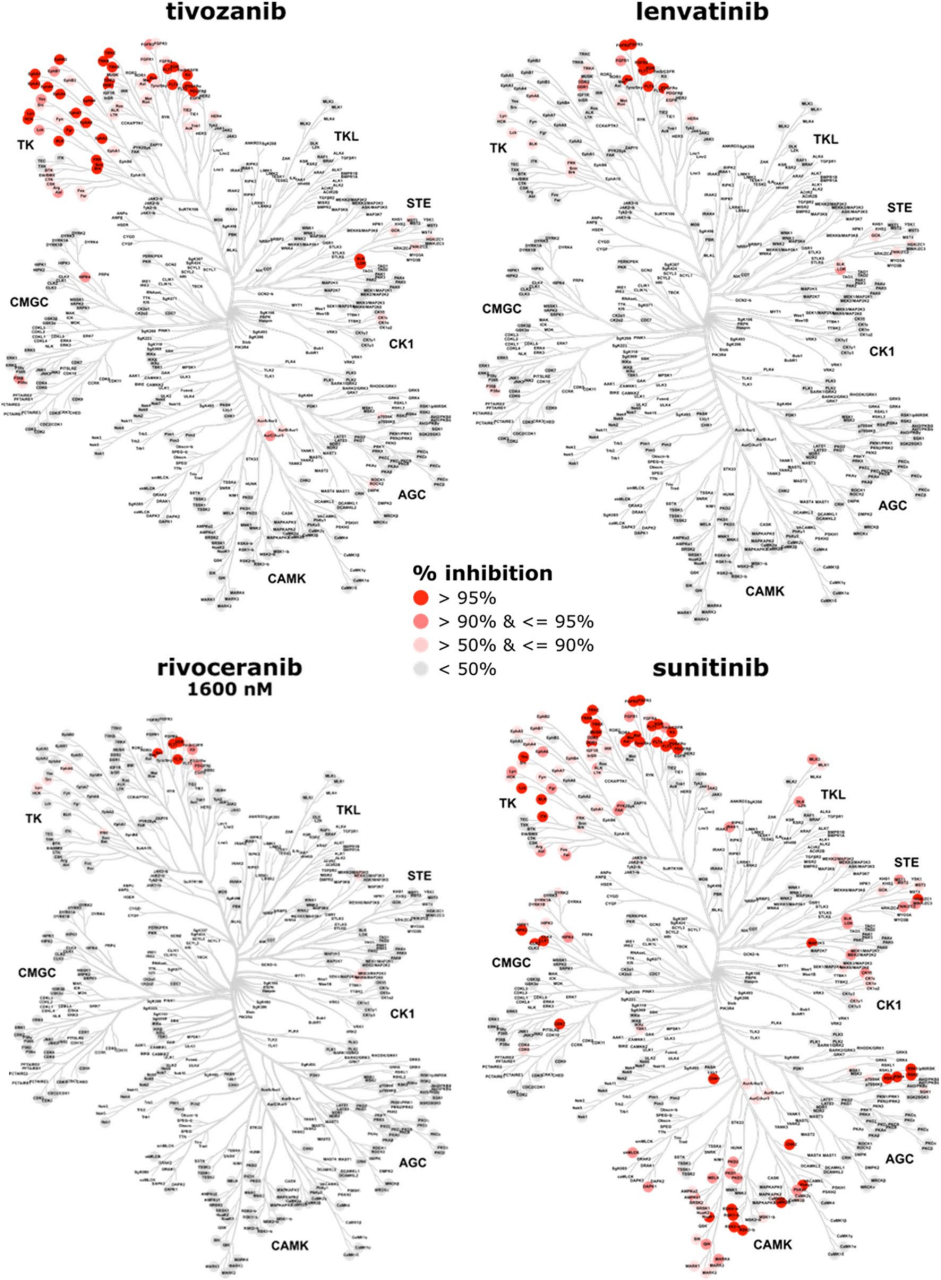
Rivoceranib is the most selective inhibitor of VEGFR2 kinase activity in the current study. The kinase activity of 270 kinases was measured in the presence of a fixed concentration of each inhibitor (1600 nM rivoceranib or 1000 nM for all reference inhibitors), and the percent inhibition values were calculated for each kinase + inhibitor pair. These values were grouped into one of the following four categories: > 95%; > 90% & ≤ 95%; > 50% & ≤ 90%; and ≤ 50% and are indicated by the node colors displayed on the kinome tree. Kinome trees shown here for comparison with rivoceranib include the most potent reference inhibitor in the VEGFR2 enzyme activity assay (tivozanib) as well as inhibitors with kinome trees most (lenvatinib) and least (sunitinib) similar to rivoceranib. The kinome trees for the full panel of reference inhibitors evaluated in this study are available in Supplemental Fig. 2

To further understand the selectivity of rivoceranib compared to the reference inhibitors on an individual kinase basis, we generated kinome trees displaying the percent inhibition of each kinase in the presence of each inhibitor. These percent inhibition values were grouped into four categories (> 95%; > 90% & ≤ 95%; > 50% & ≤ 90%; and ≤ 50%) and mapped to corresponding nodes on the kinome tree. Rivoceranib demonstrated 96.1% and 100.8% inhibition of VEGFR2 at 160 nM and 1600 nM, respectively, with greater than 90% inhibition of VEGFR family members VEGFR1/FLT1 (93.3% and 99.5%) and VEGFR3/FLT4 (92.9% and 99.3%) as well as RET (71.7% and 97.9%), PDGFRβ (62.1% and 94.8%), and KIT (47.3% and 92.6%) at the two rivoceranib concentrations, respectively (Fig. 4, Supplemental Table 3, and Supplemental Fig. 1). Compared with rivoceranib, all reference inhibitors tested demonstrated activity against a broader array of kinases (Fig. 4 and Supplemental Fig. 2). Tivozanib, the most potent VEGFR2 inhibitor in the analyses described above, displayed greater than 50% inhibitory activity against more than 70 additional kinases beyond VEGFR2 (Fig. 4). Similarly, sunitinib inhibited 125 additional kinases by > 50% and was identified as the least selective inhibitor in this study. Lenvatinib demonstrated the most similar selectivity to rivoceranib in terms of the number of additional kinases affected, with 31 kinases inhibited by 50% or more in the presence of lenvatinib. The profiles of kinases inhibited by each reference inhibitor can be found in additional kinome trees in Supplemental Fig. 2. Together, these data demonstrate that rivoceranib is the most selective inhibitor of VEGFR2 evaluated in this study.

## Discussion

With the continuing expansion of therapeutic options for targeting VEGFR2 in cancer, an understanding of the potency and selectivity of available and investigational agents is necessary to inform clinical decision-making. Here, we performed comparative biochemical analyses of the inhibitory effects of rivoceranib and a panel of 10 FDA-approved small molecules with known activity against VEGFR2. An approximate 30-fold difference in the potency of VEGFR2 kinase inhibition was found between the most and least potent inhibitors, and rivoceranib potency was within the range of the panel of reference inhibitors. Substantial variation in the selectivity of the inhibitors tested was also observed, and rivoceranib was identified as the most selective VEGFR2 inhibitor compared with the reference inhibitors.

The K_D_ for binding of rivoceranib to VEGFR2 was approximately 2 nM in this study. This low nanomolar binding affinity of rivoceranib for VEGFR2 is consistent with the low nanomolar IC_50_ for rivoceranib-mediated inhibition of VEGFR2 kinase activity [17].

Consistent with previous reports, rivoceranib demonstrated inhibition of VEGFR2 kinase activity within the range of that of currently available TKIs [17, 25]. The IC_50_ values for the reference inhibitors determined in this study are generally consistent with reported values (Supplemental Table 4), although the absolute values vary between studies. However, assay conditions used in these other studies may strongly differ, whereas we used the same panel and assay conditions for all VEGFR2 inhibitors [21, 22]. Despite the differences in absolute values, the relative potencies among the reference inhibitors are comparable to the results of this study.

In this study, rivoceranib demonstrated VEGFR2 kinase inhibition with an IC_50_ of 16 nM, while a previous study has indicated an IC_50_ value as low as 1 nM [17]. In this earlier study, a lower ATP concentration (10 µM) was used compared with our assay (75 μM). Assays in both studies were performed at an ATP concentration at or close to *K*_M,ATP_, indicating that the different IC_50_ values reflect subtle differences in the recombinant proteins and reaction conditions used in the assays. Notably, following oral administration of a clinically relevant dose of rivoceranib (250 mg QD), the unbound maximum concentration is expected to be within the range of 5 to 10 nM (data not presented), which is greater than the IC_50_ value for rivoceranib and lower than the selected concentrations of rivoceranib evaluated in this study (i.e., 160 nM and 1600 nM). Our analysis of the inhibitory profiles of rivoceranib and the reference inhibitors against a panel of 270 known kinases detected substantial variation in selectivity between compounds, with rivoceranib identified as the most selective inhibitor of VEGFR2. These differences in selectivity between compounds within a similar range of potency of VEGFR2 kinase inhibition are clinically relevant, as toxicities associated with available VEGFR2 inhibitors are thought to be due in part to their inhibitory effects against kinases outside of the VEGFR family. With the dramatic improvement in selectivity seen with rivoceranib, more effective targeting of VEGFR2 may be achieved due to an ability to deliver higher therapeutic doses with fewer off-target toxicities. This improved ability to reach higher drug concentrations would potentially result in greater anti-tumor efficacy as well as a capacity to achieve adequate concentrations of the drug in sites with limited drug penetration, such as brain metastases [26]. Furthermore, the improved VEGFR2 targeting with rivoceranib has implications for combination therapy approaches, particularly in settings in which the toxicities associated with other TKIs have limited dosing or delivery of the agents. The ability to selectively target VEGFR2 also supports the rationale for combination approaches based on drug mechanism of action; for example, VEGF signaling has been shown to suppress T cell priming and induce T cell exhaustion, providing rationale for anti-VEGFR plus immune checkpoint blockade combinations, as both classes of drugs may result in increasing the anti-cancer T cell efficacy [27]. Rivoceranib is currently under investigation in combination with the anti-PD-1 monoclonal antibody camrelizumab as first-line treatment for hepatocellular carcinoma (HCC), and clinical benefit has been observed in a randomized phase 3 trial in HCC [28]. Rivoceranib is also under ongoing investigation as monotherapy or in combination with chemotherapy in several other tumor types.

Comparison of the selectivity profile of the inhibitors included in our study may also inform personalized treatment selection in the context of molecular or biomarker assessment of the patient’s tumor, enabling selection of the drug most closely matched to the individual tumor*.* While the biochemical characteristics observed in our study should be confirmed in a cellular context, our data represent a foundation for understanding potential clinical differences in efficacy and toxicities between approved agents.

Together, our comparative biochemical analyses highlight the substantial variation in selectivity among inhibitors within a similar range of potency of VEGFR2 kinase inhibition. Rivoceranib, as the most selective inhibitor of VEGFR2, represents an attractive option for improved VEGFR2 targeting in cancer.

## Supplementary Information

Below is the link to the electronic supplementary material.

Supplementary file1 (PDF 2604 KB)

## Data Availability

The data generated in this study are available in the manuscript and supplemental methods.

## References

[1] Hanahan D, Weinberg RA (2011). Hallmarks of cancer: the next generation. Cell.

[2] Liu Y, Li Y, Wang Y, Lin C, Zhang D, Chen J (2022). Recent progress on vascular endothelial growth factor receptor inhibitors with dual targeting capabilities for tumor therapy. J Hematol Oncol.

[3] Saman H, Raza SS, Uddin S, Rasul K (2020). Inducing angiogenesis, a key step in cancer vascularization, and treatment approaches. Cancers (Basel).

[4] Apte RS, Chen DS, Ferrara N (2019). VEGF in signaling and disease: beyond discovery and development. Cell.

[5] Elebiyo TC, Rotimi D, Evbuomwan IO, Maimako RF, Iyobhebhe M, Ojo OA (2022). Reassessing vascular endothelial growth factor (VEGF) in anti-angiogenic cancer therapy. Cancer Treat Res Commun..

[6] Takahashi R, Tanaka S, Kitadai Y, Sumii M, Yoshihara M, Haruma K, Chayama K (2003). Expression of vascular endothelial growth factor and angiogenesis in gastrointestinal stromal tumor of the stomach. Oncology.

[7] Cohen MH, Gootenberg J, Keegan P, Pazdur R (2007). FDA drug approval summary: bevacizumab plus FOLFOX4 as second-line treatment of colorectal cancer. Oncologist.

[8] AVASTIN. Prescribing Information. Genentech. Updated September 2022. Accessed October 12, 2022. https://www.gene.com/download/pdf/avastin_prescribing.pdf

[9] Casak SJ, Fashoyin-Aje I, Lemery SJ, Zhang L, Jin R, Li H (2015). FDA approval summary: ramucirumab for gastric cancer. Clin Cancer Res.

[10] Choucair K, Kamran S, Saeed A (2021). Clinical evaluation of ramucirumab for the treatment of hepatocellular carcinoma (HCC): place in therapy. Onco Targets Ther.

[11] Zheng H, Qin Z, Qiu X, Zhan M, Wen F, Xu T (2020). Cost-effectiveness analysis of ramucirumab treatment for patients with hepatocellular carcinoma who progressed on sorafenib with α-fetoprotein concentrations of at least 400 ng/ml. J Med Econ.

[12] Al-Sanea MM, Chilingaryan G, Abelyan N, Sargsyan A, Hovhannisyan S, Gasparyan H (2021). Identification of novel potential VEGFR2 inhibitors using a combination of computational methods for drug discovery. Life (Basel).

[13] Li J, Qin S, Xu J, Guo W, Xiong J, Bai Y (2013). Apatinib for chemotherapy-refractory advanced metastatic gastric cancer: results from a randomized, placebo-controlled, parallel-arm, phase II trial. J Clin Oncol.

[14] Li J, Qin S, Xu J, Xiong J, Wu C, Bai Y (2016). Randomized, Double-Blind, Placebo-Controlled Phase III Trial of Apatinib in Patients With Chemotherapy-Refractory Advanced or Metastatic Adenocarcinoma of the Stomach or Gastroesophageal Junction. J Clin Oncol.

[15] Hu X, Zhang J, Xu B, Jiang Z, Ragaz J, Tong Z (2014). Multicenter phase II study of apatinib, a novel VEGFR inhibitor in heavily pretreated patients with metastatic triple-negative breast cancer. Int J Cancer.

[16] Rong X, Liu H, Yu H, Zhao J, Wang J, Wang Y (2022). Efficacy of apatinib combined with FOLFIRI in the first-line treatment of patients with metastatic colorectal cancer. Invest New Drugs.

[17] Tian S, Quan H, Xie C, Guo H, Lü F, Xu Y, Li J, Lou L (2011). YN968D1 is a novel and selective inhibitor of vascular endothelial growth factor receptor-2 tyrosine kinase with potent activity in vitro and in vivo. Cancer Sci.

[18] Mi YJ, Liang YJ, Huang HB, Zhao HY, Wu CP, Wang F (2010). Apatinib (YN968D1) reverses multidrug resistance by inhibiting the efflux function of multiple ATP-binding cassette transporters. Cancer Res.

[19] Liu X, Zheng Q, Yu Q, Hu Y, Cheng Y, Shao Z (2021). Apatinib regulates the growth of gastric cancer cells by modulating apoptosis and autophagy. Naunyn Schmied Arch Pharmacol.

[20] Kang YK, Ryu MH, Park SR, Hong YS, Choi CM, Kim TW (2016). A phase II study of apatinib, a highly selective inhibitor of VEGFR-2, in patients with metastatic solid tumors without standard treatment options. Ann Oncol.

[21] Willemsen-Seegers N, Uitdehaag JCM, Prinsen MBW, de Vetter JRF, de Man J, Sawa M, Kawase Y, Buijsman RC, Zaman GJR (2017). Compound selectivity and target residence time of kinase inhibitors studied with surface plasmon resonance. J Mol Biol.

[22] Uitdehaag JC, de Roos JA, van Doornmalen AM, Prinsen MB, de Man J, Tanizawa Y (2014). Comparison of the cancer gene targeting and biochemical selectivities of all targeted kinase inhibitors approved for clinical use. PLoS One..

[23] Uitdehaag JCM, Kooijman JJ, de Roos JADM, Prinsen MBW, Dylus J, Willemsen-Seegers N, Kawase Y (2019). Combined cellular and biochemical profiling to identify predictive drug response biomarkers for kinase inhibitors approved for clinical use between 2013 and 2017. Mol Cancer Ther.

[24] Metz KS, Deoudes EM, Berginski ME, Jimenez-Ruiz I, Aksoy BA, Hammerbacher J, Gomez SM, Phanstiel DH (2018). Coral: clear and customizable visualization of human kinome data. Cell Syst.

[25] Bhargava P, Robinson MO (2011). Development of second-generation VEGFR tyrosine kinase inhibitors: current status. Curr Oncol Rep.

[26] Gerritse SL, Janssen JBE, Labots M, de Vries R, Rudek M, Carducci M (2021). High-dose administration of tyrosine kinase inhibitors to improve clinical benefit: a systematic review. Cancer Treat Rev..

[27] Lee WS, Yang H, Chon HJ, Kim C (2020). Combination of anti-angiogenic therapy and immune checkpoint blockade normalizes vascular-immune crosstalk to potentiate cancer immunity. Exp Mol Med.

[28] Qin S, Chan LS, Gu S, Bai Y, Ren Z, Lin X (2022). LBA35 Camrelizumab (C) plus rivoceranib (R) vs sorafenib (S) as first-line therapy for unresectable hepatocellular carcinoma (uHCC): A randomized, phase III trial. Ann Oncol.

