# Supplementary material for: Comparative biochemical kinase activity analysis identifies rivoceranib as a highly selective VEGFR2 inhibitor
Source: Cancer Chemother Pharmacol. 2023 May 6;91(6):491–9. doi: 10.1007/s00280-023-04534-7 (PMC10192158; doi:10.1007/s00280-023-04534-7)
Supplement: Supplementary file 1 — Supplementary file1 (PDF 2604 KB) [file 280_2023_4534_MOESM1_ESM.pdf]

Supplemental Methods

Kinases included in enzyme activity assays and mapped to kinome trees

The following target kinases were evaluated in the enzyme activity assays:

Tyrosine kinases: ABL, ACK, ALK, ARG, AXL, BLK, BMX, BRK, BTK, CSK, DDR1, DDR2, EGFR, EPHA1, EPHA2, EPHA3, EPHA4, EPHA5, EPHA6, EPHA7, EPHA8, EPHB1, EPHB2, EPHB3, EPHB4, FAK, FER, FES, FGFR1, FGFR2, FGFR3, FGFR4, FGR, FLT1, FLT3, FLT4, FMS, FRK, FYN[isoform a], FYN[isoform b], HCK, HER2, HER4, IGF1R, INSR, IRR, ITK, JAK1, JAK2, JAK3, KDR, KIT, LCK, LTK, LYNa, LYNb, MER, MET, MUSK, PDGFRα, PDGFRβ, PYK2, RET, RON, ROS, SRC, SRM, SYK, TEC, TIE2, TNK1, TRKA, TRKB, TRKC, TXK, TYK2, TYRO3, YES, ZAP70.

Serine/Threonine kinases: AKT1, AKT2, AKT3, AMPKα1/β1/γ1, AMPKα2/β1/γ1, AurA, AurA/TPX2, AurB/INCENP, AurC, BRAF_Cascade, BRSK1, BRSK2, BUB1/BUB3, CaMK1α, CaMK1δ, CaMK2α, CaMK2β, CaMK2γ, CaMK2δ, CaMK4, CDC2/CycB1, CDC7/ASK, CDK2/CycA2, CDK2/CycE1, CDK3/CycE1, CDK4/CycD3, CDK5/p25, CDK6/CycD3, CDK7/CycH/MAT1, CDK9/CycT1, CGK2, CHK1, CHK2, CK1α, CK1γ1, CK1γ2, CK1γ3, CK1δ, CK1ε, CK2α1/β, CK2α2/β, CLK1, CLK2, CLK3, COT_Cascade, CRIK, DAPK1, DCAMKL2, DLK_Cascade, DYRK1A, DYRK1B, DYRK2, DYRK3, EEF2K, Erk1, Erk2, Erk5, GSK3α, GSK3β, Haspin, HGK, HIPK1, HIPK2, HIPK3, HIPK4, HPK1, IKKα, IKKβ, IKKε, IRAK1, IRAK4, JNK1, JNK2, JNK3, LATS2, LOK, MAP2K1_Cascade, MAP2K2_Cascade, MAP2K3_Cascade, MAP2K4_Cascade, MAP2K5_Cascade, MAP2K6_Cascade, MAP2K7_Cascade, MAP3K1_Cascade, MAP3K2_Cascade, MAP3K3_Cascade, MAP3K4_Cascade, MAP3K5_Cascade, MAP4K2, MAPKAPK2, MAPKAPK3, MAPKAPK5, MARK1, MARK2, MARK3, MARK4, MELK, MINK, MLK1_Cascade, MLK2_Cascade, MLK3_Cascade, MNK1, MNK2, MOS_Cascade, MRCKα, MRCKβ, MSK1, MSK2, MSSK1, MST1, MST2, MST3, MST4, NDR1, NDR2, NEK1, NEK2, NEK4, NEK6, NEK7, NEK9, NIM1K, NuaK1, NuaK2, p38α, p38β, p38γ, p38δ, p70S6K, p70S6Kβ, PAK1, PAK2, PAK4, PAK5, PAK6, PASK, PBK, PDHK2, PDHK4, PDK1, PEK, PGK, PHKG1, PHKG2, PIM1, PIM2, PIM3, PKACα, PKACβ, PKACγ, PKCα, PKCβ1, PKCβ2, PKCγ, PKCδ, PKCε, PKCζ, PKCη, PKCθ, PKCι, PKD1, PKD2, PKD3, PKN1, PKR, PLK1, PLK2, PLK3, PRKX, QIK, RAF1_Cascade, ROCK1, ROCK2, RSK1, RSK2, RSK3, RSK4, SGK, SGK2, SGK3, SIK, skMLCK, SLK, SRPK1, SRPK2, TAOK2, TBK1, TNIK, TSSK1, TSSK2, TSSK3, WNK1, WNK2, WNK3.

Other kinases: SPHK1, SPHK2.

Supplemental Figures and Tables

Supplemental Figure 1: Rivoceranib-mediated inhibition across kinome tree at 10-fold dose difference

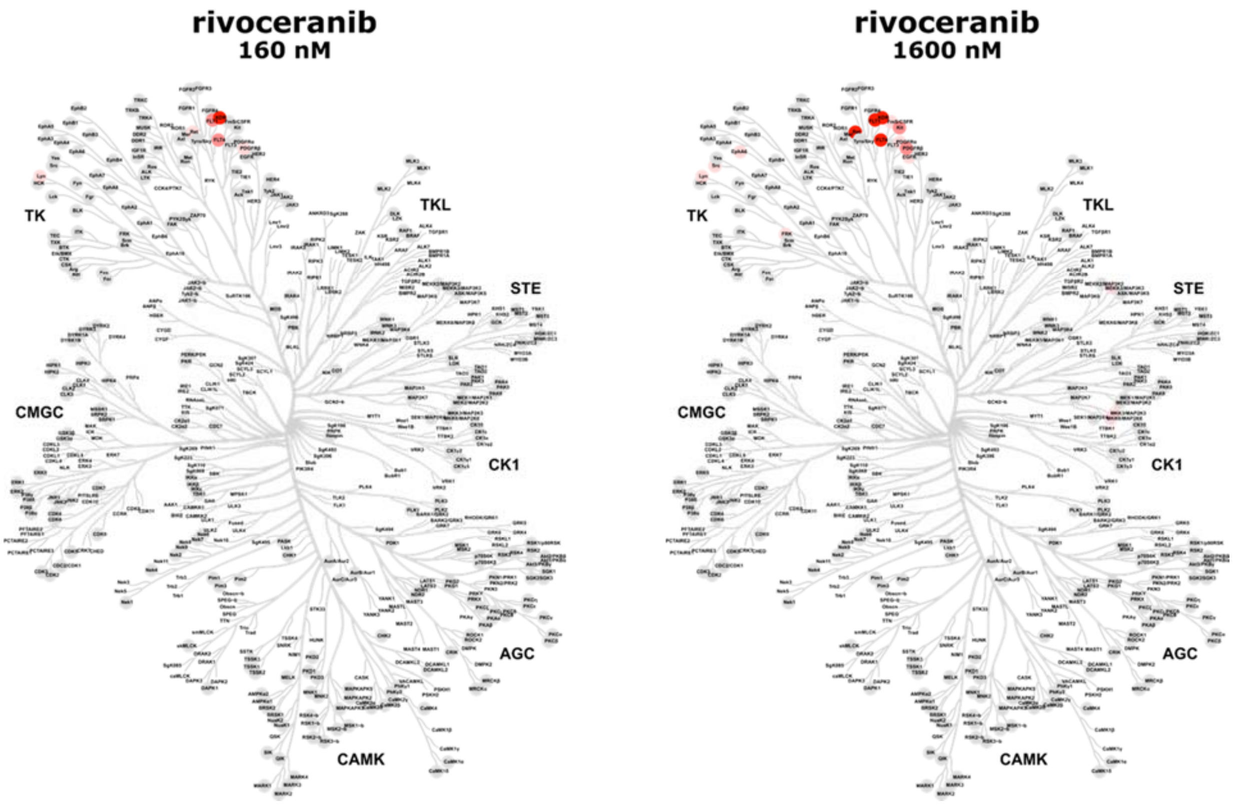

Supplemental Figure 1: Rivoceranib-mediated inhibition across kinome tree at 10-fold dose difference. The kinase activity of 270 kinases was tested in the presence of 160 nM or 1600 nM rivoceranib, and the percentage inhibition of each kinase was assigned to one of the following four categories: : > 95%; > 90% & ≤95%; > 50% & ≤ 90%; and ≤ 50%, indicated by the node colors displayed on the kinome tree.

Supplemental Figure 2: Comparison of selectivity of all 10 FDA-approved inhibitors included in this study.

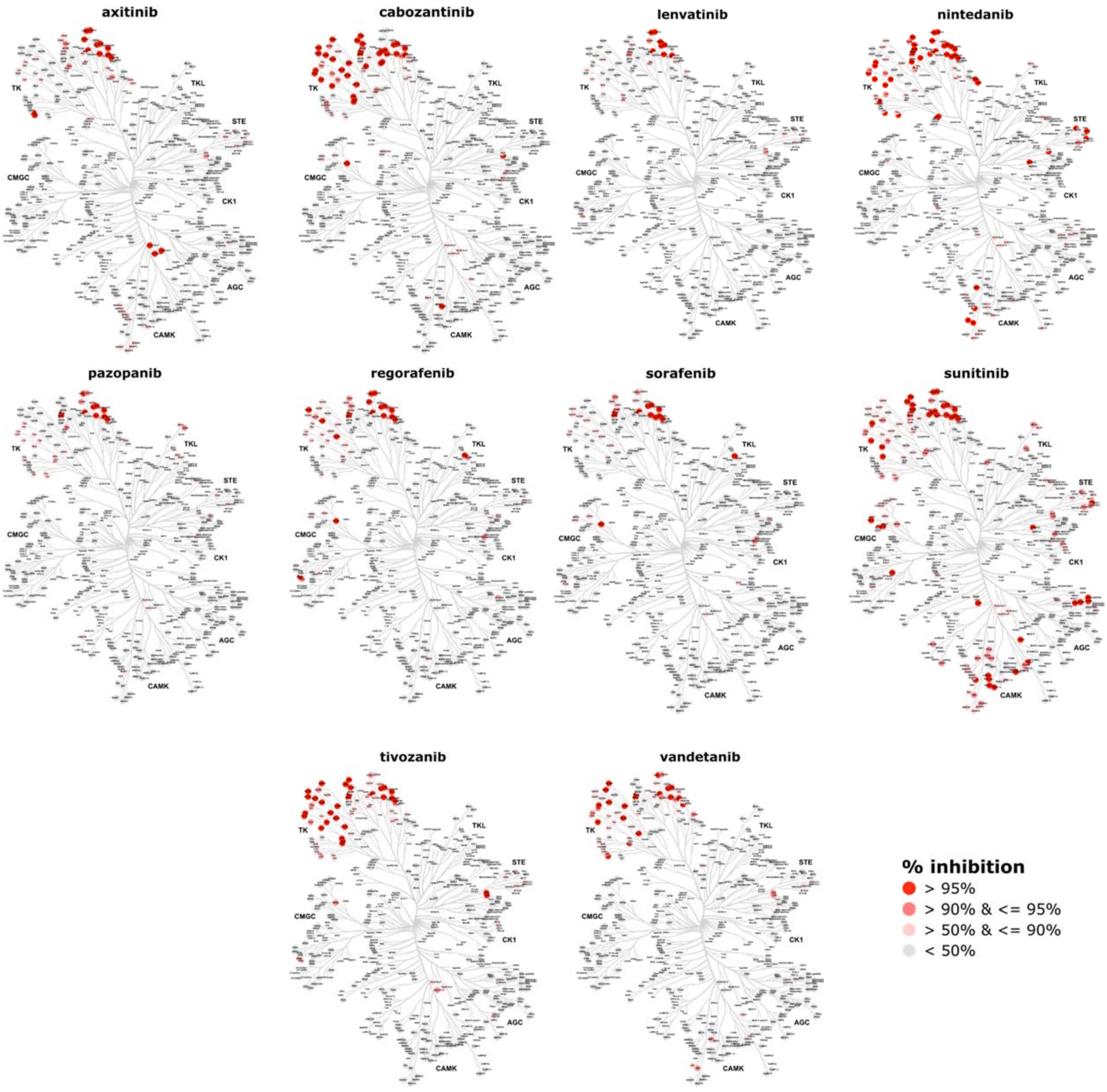

Supplemental Figure 2: Comparison of selectivity of all 10 FDA-approved inhibitors included in this study. The kinase activity of 270 kinases was tested in the presence of 1000 nM of the specified inhibitor, and the percentage inhibition of each kinase was assigned to one of the following four categories: > 95%; > 90% & ≤95%; > 50% & ≤ 90%; and ≤ 50%, indicated by the node colors displayed on the kinome tree.

Supplemental Table 1: Inhibitor concentration ranges in dose-response assays

**Inhibitor**	**Maximum concentration (μM)**	**Minimum concentration (nM)**
rivoceranib	100	3
axitinib	10	0.3
cabozantinib	10	0.3
lenvatinib	1	0.03
nintedanib	1	0.03
pazopanib	10	0.3
regorafenib	10	0.3
sorafenib	10	0.3
sunitinib	10	0.3
tivozanib	1	0.03
vandetanib	10	0.3

Supplemental Table 2: Kinetic parameters of rivoceranib binding to VEGFR2 determined by SPR

**Experiment***	**k_a (1/Ms)**	**k_d (1/s)**	**K_D (M)**
1a	3.03E+05	5.57E-04	1.84E-09
1b	4.45E+05	4.45E-04	1.00E-09
2a	6.59E+05	2.20E-03	3.34E-09
2b	2.53E+05	7.75E-04	3.06E-09
Geometric average	3.87E+05	8.07E-04	2.08E-09

*1 and 2 represent two independent experiments (experimental replicates), in which kinetic parameters were determined in duplicate (technical replicates, a and b).

Supplemental Table 3: Percent inhibition of kinases inhibited >90% by rivoceranib at 160 nM or 1600 nM.

Kinase	Rivoceranib (160 nM)	Rivoceranib (1600 nM)
VEGFR2/KDR	96.12	100.79
VEGFR1/FLT1	93.27	99.53
VEGFR3/FLT4	92.90	99.33
RET	71.72	97.89
PDGFRβ	62.11	94.80
KIT	47.30	92.58

Supplemental Table 4: Reported VEGFR2 IC_{50} values for reference inhibitors from published literature.

Inhibitor	Reported IC_{50} values	IC_{50} value in this study	Reference
Axitinib	0.2 nM	1.1 nM	Gross-Goupil M, François L, Quivy A, Ravaud A. Axitinib: a review of its safety and efficacy in the treatment of adults with advanced renal cell carcinoma. Clin Med Insights Oncol. 2013 Oct 29;7:269-77. doi: 10.4137/CMO.S10594.
Cabozantinib	0.35 nM	2.5 nM	Yakes FM, Chen J, Tan J, Yamaguchi K, Shi Y, Yu P, Qian F, Chu F, Bentzien F, Cancilla B, Orf J, You A, Laird AD, Engst S, Lee L, Lesch J, Chou YC, Joly AH. Cabozantinib (XL184), a novel MET and VEGFR2 inhibitor, simultaneously suppresses metastasis, angiogenesis, and tumor growth. Mol Cancer Ther. 2011 Dec;10(12):2298-308. doi: 10.1158/1535-7163.MCT-11-0264.
Lenvatinib	4 nM	1.3 nM	Brody T. FDA's Drug Review Process and the Package Label: Strategies for Writing Successful FDA Submissions. 2018. https://doi.org/10.1016/C2017-0-01355-1.
Nintedanib	21 nM	1.4 nM	Roth GJ, Binder R, Colbatzky F, Dallinger C, Schlenker-Herceg R, Hillberg F, Wollin S-L, Raiser R. Nintedanib: From Discovery to the Clinic J. Med. Chem. 2015;(58):1053−1063. dx.doi.org/10.1021/jm501562a.
Pazopanib	30 nM	7.1 nM	Gross-Goupil M, François L, Quivy A, Ravaud A. Axitinib: a review of its safety and efficacy in the treatment of adults with advanced renal cell carcinoma. Clin Med Insights Oncol. 2013 Oct 29;7:269-77. doi: 10.4137/CMO.S10594.
Regorafenib	4 nM	16 nM	Wilhelm SM, Dumas J, Adnane L, Lynch M, Carter CA, Schütz G, Thierauch KH, Zopf D. Regorafenib (BAY 73-4506): a new oral multikinase inhibitor of angiogenic, stromal and oncogenic receptor tyrosine kinases with potent preclinical antitumor activity. Int J Cancer. 2011 Jul 1;129(1):245-55. doi: 10.1002/ijc.25864.
Sorafenib	90 nM	29 nM	Gross-Goupil M, François L, Quivy A, Ravaud A. Axitinib: a review of its safety and efficacy in the treatment of adults with advanced

			renal cell carcinoma. Clin Med Insights Oncol. 2013 Oct 29;7:269-77. doi: 10.4137/CMO.S10594.
Sunitinib	10 nM	20 nM	Gross-Goupil M, François L, Quivy A, Ravaud A. Axitinib: a review of its safety and efficacy in the treatment of adults with advanced renal cell carcinoma. Clin Med Insights Oncol. 2013 Oct 29;7:269-77. doi: 10.4137/CMO.S10594.
Tivozanib	0.16 nM	0.95 nM	Yalcin S, Lacin S. Impact of tivozanib on patient outcomes in treatment of advanced renal cell carcinoma. Cancer Manag Res. 2019 Aug 16;11:7779-7785. doi: 10.2147/CMAR.S206105.
Vandetanib	40 nM	4.5 nM	Brody T. FDA's Drug Review Process and the Package Label: Strategies for Writing Successful FDA Submissions. 2018. https://doi.org/10.1016/C2017-0-01355-1.
